# Patterns and predictors of statin prescription in patients with type 2 diabetes

**DOI:** 10.1186/1475-2840-8-25

**Published:** 2009-05-13

**Authors:** Heiner K Berthold, Ioanna Gouni-Berthold, Michael Böhm, Wilhelm Krone, Kurt P Bestehorn

**Affiliations:** 1 Charité University Medicine Berlin, Virchow Clinic Campus, Lipid Clinic at the Interdisciplinary Metabolism Center, Berlin, Germany; 2 University of Cologne, Department of Internal Medicine II, Cologne, Germany; 3 University of Homburg, Department of Internal Medicine III, Homburg/Saar, Germany; 4 MSD Sharp & Dohme GmbH, Haar, Germany

## Abstract

**Background:**

The benefit of statins for prevention of cardiovascular events in type 2 diabetes is established, but a gap exists between guideline recommendations and clinical practice. The aim of the study was to identify patient-related factors predicting statin prescription.

**Methods:**

We assessed the quality of care in 51,640 patients with type 2 diabetes in a German diabetes registry. Patients were stratified according to primary and secondary prevention. Five-year risk for cardiovascular events was calculated in primary prevention patients. A multivariate adjusted logistic regression model was constructed to determine which parameters influenced statin prescription.

**Results:**

34% had established atherosclerotic disease and 25.5% received a statin. Prescription was significantly higher in the secondary compared to the primary prevention group (38.1% [95% CI 37.4–38.9%] vs. 18.5% [95% CI 18.0–19.0%], respectively). In primary prevention the odds for statin prescription increased with estimated cardiovascular risk (OR 1.17 per 5% increase in 5-year risk, 95% CI 1.11–1.22). Positive predictors for statin prescription were secondary prevention, hypertension, former smoking, baseline LDL-cholesterol, and microalbuminuria. The odds of receiving a statin had an inverted U-shaped relation with age (nadir, 66 years), age at first diagnosis of diabetes (nadir, 56 years), and body mass index (nadir, 32 kg/m^2^). The model predicted prescription in 70% of the patients correctly.

**Conclusion:**

The majority of patients with type 2 diabetes are not receiving statins. The predominant factors determining statin prescription are the patient's prevention status and, in primary prevention, estimated cardiovascular risk. The results suggest that although physicians are aware of the general concept of cardiovascular risk, they fail to consistently implement guidelines.

## Background

Patients with diabetes have a substantially increased risk of atherosclerotic vascular disease [1]. A recent meta-analysis examined whether statins (HMG-CoA reductase inhibitors) are as beneficial in preventing cardiovascular events in patients with diabetes as they are in those without, and found that in patients with diabetes there was a 9% proportional reduction in all-cause mortality per mmol/l (~40 mg/dl) reduction on LDL cholesterol, a reduction similar to the 13% reduction seen in patients without the disease [2]. There were significant reductions in the numbers of fatal and non-fatal myocardial infarctions, coronary revascularisations, and strokes. Moreover, the relative risk reduction was independent of previous history of vascular disease and of baseline subject characteristics.

Current type 2 diabetes guidelines issued by European and American scientific societies recommend lipid-lowering treatment with statins in order to reach the LDL-C target levels of < 100 mg/dl or of < 70 mg/dl in individuals with coexisting cardiovascular disease (CVD) [3-5]. However, studies suggest that large discrepancies exist between treatment targets and clinical reality. We have recently shown that the proportion of patients with type 2 diabetes in Germany receiving a statin is low, namely only 25% [6]. Moreover, only about 6% of these patients reach all three lipoprotein targets (LDL-C < 100 mg/dl, triglycerides < 150 mg/dl and HDL > 40 or 50 mg/dl in men or women, respectively) [7]. Similarly, reports from other European countries [8,9] and the United States [10] show that not all eligible patients receive cholesterol-lowering therapy.

The reasons behind statin undertreatment are unclear. Statins are available in generic form and they are affordable. However, while in Germany costs for reimbursement are covered by health insurers, physicians' prescribing habits are constrained by a pre-defined overall drug budget. Professional bodies in Germany are reluctant recommending statin therapy for all diabetic patients (e. g. [11]) despite unequivocal evidence of their efficacy from numerous randomized trials and meta-analyses [12-14]. While there is an ongoing debate as to whether diabetes should be considered a coronary heart disease (CHD) equivalent [15], the National Cholesterol Education Programme defined the status of diabetes mellitus already in 2004 as a CHD risk equivalent [16].

The main goal of the present study was to identify patient characteristics predictive of who would receive a statin (or not). For pragmatic reasons, we decided to stratify the patients in bivariate analyses according to primary and secondary prevention before constructing a multivariate logistic regression model including the prevention status. We used the DUTY diabetes registry [6,17] with > 50,000 patients from all over Germany.

## Methods

### Study design and subjects

The DUTY registry (Diabetes mellitus needs unrestricted evaluation of patient data to yield treatment progress) is a cross-sectional study in outpatients with type 2 diabetes. The study protocol was approved by the Ethics Committee of the Bavarian Chamber of Physicians. The study design has been published elsewhere [18]. In short, between February 2002 and November 2003 office-based physicians in Germany were approached to participate in the study and to recruit 20 consecutive diabetic patients. Reports of 59,075 patients from 3213 physicians were received. Of these patients, 89.8% had type 2 diabetes, 5.7% type 1, and in 4.5% the type of diabetes was not identified. For the present evaluation, only patients with type 2 diabetes were considered. Moreover, only data sets where patient gender could be identified were considered. Thus, data of 51,640 patient data sets were analyzed.

Main outcome measure was receiving a statin prescription. We investigated in bivariate analyses factors associated with statin prescription in subjects stratified according to whether they had a history of CHD, stroke, or peripheral arterial occlusive disease (secondary prevention), and in subjects presumed free of atherosclerotic complications (primary prevention).

### Definition of parameters

Diabetes was defined by the treating physician. Hypertension was defined by the presence of antihypertensive drug therapy or by a blood pressure reading of ≥ 140 mmHg systolic or ≥ 90 mmHg diastolic. Lipoproteins, HbA1c, and albumin in urine were determined with routine laboratory methods. Microalbuminuria was defined as albumin excretion in urine ≥ 20 mg/l. Smoking status was documented as self-reported current smoking, former smoking, and never smoking. Glomerular filtration rate was estimated using the Modification of Diet in Renal Disease formula [19].

In the primary prevention group, 5-year risk estimates for the prediction of cardiovascular disease were calculated using the equations developed with the data of the Swedish National Diabetes Registry according to Cederholm et al. [20]. The risk equation contains the following parameters: age at onset of diabetes, duration of diabetes, HbA1c, body mass index (BMI), systolic blood pressure, sex, smoking status, and antihypertensive and lipid-lowering medications.

Lipoprotein concentrations are an important determinant of prescribing a statin. Since in a cross-sectional study there are no baseline 'untreated' levels available, we modelled baseline LDL cholesterol levels in the subjects receiving lipid-lowering drug therapy. The measured LDL levels were corrected assuming a 15% reduction. This effect size was calculated based on the following assumptions: We used the statins prescribed in Germany in 2002 and their daily defined doses [21] and calculated their expected LDL-lowering effect. Statin medication possession ratio (i.e. the number of doses dispensed in relation to the dispensing period) and adherence to treatment (i.e. doses taken in relation to what was prescribed) were assumed to be 50 to 75% and 50%, respectively, as previously described [22,23].

### Statistical methods

**Figure 1:**
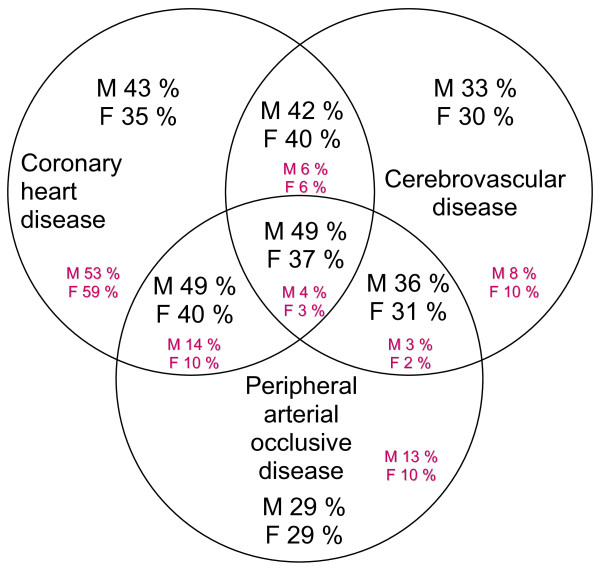
**Prevalence of the different atherosclerotic disease entities (small figures) and respective statin prescription frequencies (large figures) for men (M) and women (F)**. Note that some patients had > 1 atherosclerotic disease manifestation sites, so the numbers may add up to > 100%.

Continuous variables are given as mean values ± standard deviations. Categorical variables are described as proportions (percentage) and 95% confidence intervals were calculated from binomial distributions. We stratified all evaluations in our first model according to primary or secondary prevention. Of note, some of the patients had more than one atherosclerotic disease entity (Figure 1).

We used bivariate analyses to examine frequency distributions and variability and we calculated odds ratios and *P *values for the main outcome measure in a 1^st ^model from simple logistic regression analyses. In a 2^nd ^model, we multiple-adjusted for parameters that were identified in the 1^st ^model. Forward and backward stepwise logistic regression using maximum likelihood-ratio statistics were used for further support of variable selection. The full model included sex, age, age at diagnosis of diabetes, BMI, smoking status, established concomitant atherosclerotic disease, hypertension, microalbuminuria, estimated glomerular filtration rate, HbA1c, baseline LDL cholesterol, HDL cholesterol, and triglycerides.

**Figure 2:**
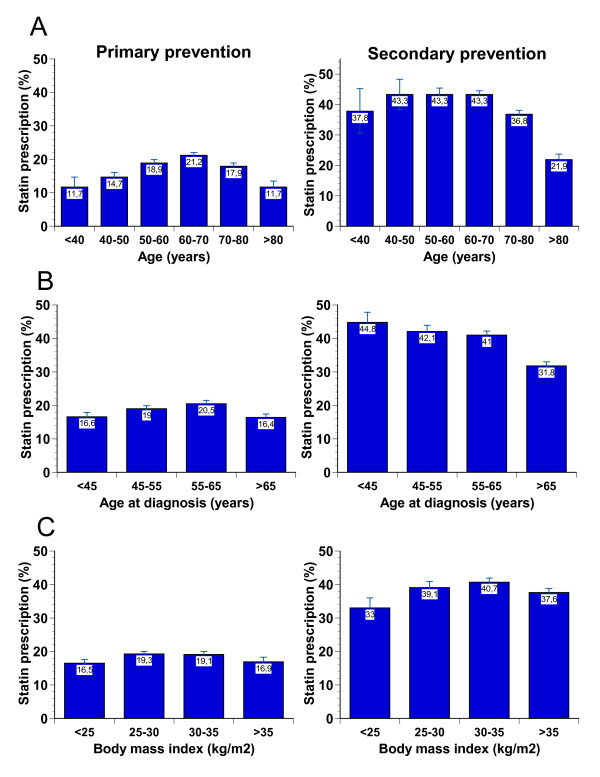
**Unadjusted statin prescription frequencies according to age (A), age at diagnosis of diabetes (B), and body mass index (C)**. Primary prevention data are shown on the left and secondary prevention on the right side. Data are proportions (%) and 95% confidence intervals for binomial distributions.

Because in 3 variables (age, age at diagnosis and BMI) in bivariate analyses statin prescription was associated with an inverted U-shape of the data (see Figure 2), we added squared terms of these variables. Due to differential prescription patterns seen in primary and secondary prevention (see Table 1), we added the interaction terms 'sex*atherosclerotic disease', 'baseline LDL cholesterol*atherosclerotic disease', and 'smoking status*atherosclerotic disease'. Other interactions were evaluated for inclusion but none was found to improve the model. Missing data on atherosclerotic disease were imputed as 'no known atherosclerotic disease'. Otherwise no missing data imputations were performed. The adequacy of the derived model equation was assessed by standard goodness-of-fit procedures. Sensitivity analysis of the model was performed by repeating the analyses using prescription of all lipid-lowering drugs therapies (e. g., fibrates) rather than the prescription of statins alone. These results were similar and the data are not shown. We used the Statistical Package for the Social Sciences Version 16.1.2 for all calculations (SPSS Inc., Munich, Germany). All statistical tests were performed two-sided and a *P*-value of < 0.05 was considered significant.

**Table 1:** Demographic and clinical characteristics in patients with and without statin prescription.*

**Characteristic**	**Statin prescription**	**No statin prescription**
Number, (%)	13,150 (25.5%)	38,490 (74.5%)
		
Men (%)	27.6	72.4
Women (%)	23.4	76.6
Age (years)	65.6 ± 9.5	65.1 ± 11.3
Age at diagnosis of diabetes (years)	58.3 ± 10.2	58.5 ± 11.2
Diabetes duration (years)	7.3 ± 6.4	6.6 ± 6.1
Body mass index (kg/m^2^)	28.9 ± 4.4	28.9 ± 4.9
		
Smoking status		
Never smoker (%)	23.6	76.4
Former smoker (%)	31.5	68.5
Current smoker (%)	24.4	75.6
		
Hypertension (%)	86.4	13.6
Systolic blood pressure (mmHg)	143 ± 17	143 ± 18
Diastolic blood pressure (mmHg)	83 ± 10	83 ± 10
		
Microalbuminuria (%)	42.3	57.7
Creatinine (mg/dl)	1.35 ± 2.51	1.31 ± 2.45
Estimated glomerular filtration rate (ml/min)	74 ± 36	76 ± 37
		
HbA1c (%)	7.3 ± 1.3	7.3 ± 1.3
Fasting plasma glucose (mg/dl)	147 ± 46	148 ± 48
		
Lipoprotein concentrations		
Total cholesterol (mg/dl)	221 ± 53	226 ± 50
LDL cholesterol (mg/dl)	131 ± 40	138 ± 38
HDL cholesterol (mg/dl)	48 ± 13	49 ± 14
Triglycerides (mg/dl)	226 ± 260	218 ± 313
		
Estimated 5-year cardiovascular risk (primary prevention)	18.3 ± 10.6	14.5 ± 10.1
Atherosclerotic complications		
Coronary heart disease (%)	43.5	56.5
Cerebrovascular disease (%)	11.0	89.0
Peripheral art. occlusive dis. (%)	15.5	84.5

*Data are means ± SD or proportions (in %).

## Results

### Patient demographics, comorbidities, and complications

Patient demographics and the presence of individual covariates are shown in Table 2. There were 52.9% of the subjects in primary prevention and 34.0% in secondary prevention (in 13.1% the prevention status could not be identified). In secondary prevention, 78.3% had CHD, 21.2% cerebrovascular disease, and 25.7% peripheral artery disease. The prevalence of the different vascular disease entities is shown in Figure 1. More than 83% in primary and > 94% in secondary prevention had hypertension. Albuminuria was present in 19.6% in primary and 39.1% in secondary prevention.

**Table 2:** Subject demographics for patients with type 2 diabetes (*n *= 51,640) in primary or secondary prevention*.

**Characteristic**	**Primary prevention**	**Secondary prevention**	**Prevention status unknown**
Number, (%)	27,322 (52.9%)	17,571 (34.0%)	6,747 (13.1%)
			
Male sex (%)	45.5%	54.2%	46.7%
Age (years)	62.9 ± 10.9	69.3 ± 9.5	64.0 ± 10.8
Age at diagnosis of diabetes (years)	57.1 ± 10.9	61.0 ± 10.5	57.6 ± 11.0
Diabetes duration (years)	5.8 ± 5.7	8.3 ± 6.8	6.3 ± 5.9
Body mass index	29.1 ± 4.9	28.5 ± 4.4	29.1 ± 4.8
			
Smoking status (%)			
Never smoker	64.8%	54.2%	52.5%
Former smoker	17.3%	27.7%	17.8%
Current smoker	17.6%	15.0%	9.0%
			
Hypertension (%)	83.3%	94.8%	84.8%
			
Microalbuminuria (>20 mg/l)	27.8%	34.9%	31.1%
			
HbA1c (%)			
<6.5%	27.3%	23.8%	26.1%
≥6.5% and <7.5%	37.0%	34.6%	35.2%
≥7.5% and <8.5%	21.1%	24.4%	22.8%
≥8.5%	13.9%	16.3%	14.5%
			
Lipoprotein concentrations (mg/dl)			
Total cholesterol			
<200	27.8%	32.4%	30.5%
≥200 and <240	35.4%	32.0%	33.2%
≥240	34.7%	32.2%	35.0%
LDL cholesterol			
<100	12.6%	16.3%	14.1%
≥100 and <130	25.0%	24.2%	24.1%
≥130 and <160	26.9%	23.6%	24.6%
≥160	22.6%	21.4%	21.8%
HDL cholesterol			
<40 (M) or <50 (F)	36.2%	39.3%	36.0%
≥40 (M) or ≥50 (F)	55.8%	53.6%	55.2%
Triglycerides			
<150	30.5%	29.0%	28.9%
≥150 and <400	50.1%	51.7%	50.2%
≥400	4.5%	4.9%	3.9%
			
On lipid-lowering medication (%)			
Any	24.0%	45.8%	31.6%
Statins	18.5%	38.1%	25.1%
Fibrates	5.8%	7.9%	7.2%
Other	0.11%	0.14%	0.10%
			
Atherosclerotic risk or disease, respectively	5-Year atherosclerotic disease risk (%)	Presence of atherosclerotic disease (%)	unknown
	<5%: 11.0%	Coronary heart disease: 78.3%	
	≥5% and <10%: 21.9%	Cerebrovascular disease: 21.2%	
	≥10% and <15%: 19.2%	Periph. art. occlussive dis.: 25.7%	
	≥15% and <20%: 14.0%		
	≥20% and <25%: 9.3%		
	≥25% and <30%: 5.7%		

	≥30%: 8.1%		

In 6747 patients (13.1%) the prevention status was unknown

*Data are means ± SD or proportions (in %).

Overall, in primary prevention 12.6% of the patients reached LDL cholesterol target levels of < 100 mg/dl and in secondary prevention 16.3%. The proportion of primary prevention patients in various classes of 5-year estimated risk can be seen in Table 2.

### Statin treatment: bivariate analyses

Table 1 shows demographic and clinical characteristics in patients with and without statin prescription. In primary prevention 24% of the patients were on any type of lipid-lowering drug therapy and 18.5% were receiving a statin, while in secondary prevention 46% were receiving any type of lipid-lowering drug therapy and 38% a statin. Fibrates were prescribed in 5.8% and 7.9% of patients, respectively. The respective statin prescription frequencies according to atherosclerotic disease manifestation site are depicted in Figure 1. Statin prescription was higher in men than in women in general and higher in patients with CHD than in patients with cerebrovascular and peripheral arterial disease. Highest prescription frequencies were observed when CHD and peripheral disease were concurrently present.

Table 3 shows bivariate analyses of factors determining statin prescription. Results are expressed in percent of the respective group receiving a statin. Odds ratios for receiving a statin and confidence intervals for binomial distributions can be obtained from the online Additional file 1.

**Table 3:** Proportions of statin prescription in bivariate analysis.

**Characteristic**	**Primary prevention**	**Secondary prevention**
	Percent statin use	Percent statin use
**Number**	*n *= 27,322 18.5%	*n *= 17,571 38.1%
		
**Sex**		
Male	18.5%	41.3%
Female	18.5%	34.4%
		
**Age (years)**		
<40	11.7%	37.8%
≥40 and <50	14.7%	43.3%
≥50 and <60	18.9%	43.3%
≥60 and <70	21.2%	43.3%
≥70 and <80	17.9%	36.8%
≥80	11.7%	21.9%
		
**Age at diagnosis of diabetes (years)**		
<45	16.6%	44.8%
≥45 and <55	19.0%	42.1%
≥55 and <65	20.5%	41.0%
≥65	16.4%	31.8%
		
**Diabetes duration (years)**		
<1	11.4%	37.9%
≥1 and <5	19.3%	39.2%
≥5 and <10	18.9%	38.0%
≥10	20.2%	37.1%
		
**Body mass index (kg/m^**2**^)**		
<25	16.5%	33.0%
≥25 and <30	19.3%	39.1%
≥30 and <35	19.1%	40.7%
≥35	16.9%	37.6%
		
**Smoking status**		
Never smoker	18.1%	35.2%
Former smoker	20.1%	44.8%
Current smoker	18.5%	36.0%
		
**Hypertension**		
no	11.7%	22.9%
yes	19.9%	39.0%
		
**HbA1c (%)**		
<6.5	17.8%	36.9%
≥6.5 and <7.5	19.2%	39.8%
≥7.5 and <8.5	18.8%	36.7%
≥8.5	17.6%	38.5%
		
**Albuminuria**		
No albuminuria	17.4%	37.4%
Albuminuria (≥20 mg/l)	19.0%	39.1%
		
**Lipoprotein concentrations (mg/dl)**		
Total cholesterol		
<200	19.9%	47.2%
≥200 and <240	16.2%	34.8%
≥240	19.7%	32.6%
LDL cholesterol		
<100	23.8%	54.9%
≥100 and <130	19.7%	42.0%
≥130 and <160	16.9%	32.0%
≥160	19.0%	30.9%
HDL cholesterol		
<40 (M) or <50 (F)	19.9%	38.2%
≥40 (M) or ≥50 (F)	19.1	39.9%
Triglycerides		
<150	9.8%	37.3%
≥150 and <400	20.0%	38.9%
≥400	22.9%	45.4%
		
**Estimated 5-year cardiovascular risk (%)**		
<5	6.6%	
≥5 and <10	13.9%	
≥10 and <15	19.7%	
≥15 and <20	23.4%	
≥20 and <25	23.9%	
≥25 and <30	26.9%	
≥30	26.9%	
		
**Atherosclerotic complications**		
Coronary heart disease		40.1%
Cerebrovascular disease		37.3%
Peripheral arterial occlusive disease		38.0%

Subjects were stratified according to primary or secondary prevention and groups were analyzed separately. Confidence intervals and odds ratios for receiving a statin can be obtained from Additional file 1.

In unadjusted analyses, women received less frequently statin prescriptions than men. In both, primary and secondary prevention, statin prescription was highest in the 6^th ^and 7^th ^decade of life but declined at higher age. The association with age showed an inverted U-shaped curve (nadir, 66 years; Figure 2A). Similarly, in primary prevention the odds of receiving a statin were lower at younger and higher age at first diagnosis of diabetes (nadir, 56 years), while in secondary prevention the highest prescription rates were seen in patients diagnosed at age < 45 years (Figure 2B). Diabetes duration had an influence on statin prescription in bivariate analyses (OR per year 1.018, 95% CI 1.015 to 1.021, *P *< 0.0001), but not in multivariate analyses. A body mass index of > 35 kg/m^2 ^but also BMI values < 25 kg/m^2 ^decreased the odds of statin prescription when compared with moderate overweight (nadir, BMI 32 kg/m^2^), both in primary and secondary prevention (Figure 2C). Former smokers had higher odds of receiving a statin when compared with current smokers and never smokers in secondary prevention. In primary prevention, prescription rates were comparable among never smokers, former smokers and current smokers. The subjects with hypertension had almost doubled odds of receiving a statin (Table 3 and Additional file 1). When albuminuria was present, the odds of receiving a statin increased in comparison to patients with no microalbuminuria (Table 3 and Additional file 1). Glycemic control, as reflected by HbA1c, had no influence and was not included in the final model. Estimated glomerular filtration rate was negatively associated with statin prescription (OR per ml/min 0.998, 95% CI 0.997 to 0.999, *P *< 0.0001) in bivariate but not in multivariate analyses. Triglycerides and HDL cholesterol concentrations were not associated with statin prescription.

**Figure 3:**
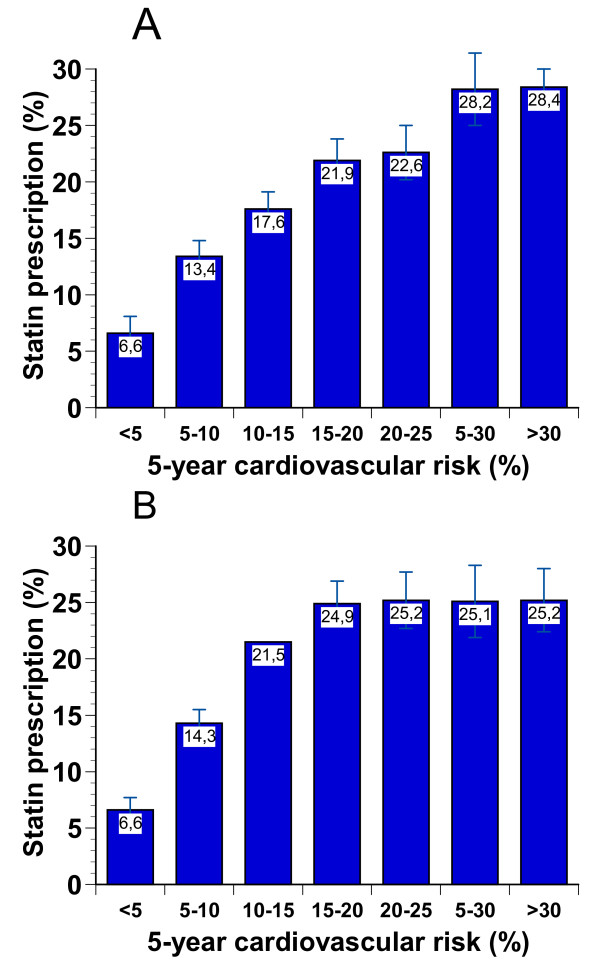
**Unadjusted statin prescription frequencies in men (A) and women (B) according to the estimated 5-year cardiovascular event risk**. Data are proportions (%) and 95% confidence intervals for binomial distributions.

In primary prevention, there was a continuous increase in prescription frequency from the lowest (< 5%) to the highest (> 30%) 5-year estimated risk groups. These data are depicted separately for men and women in Figure 3.

Table 4 shows the results when simulating baseline LDL level conditions. Prescription rates were highest in the groups of LDL cholesterol ≥160 mg/dl in both, primary and secondary prevention, but odds ratios were more pronounced in primary prevention.

**Table 4:** Baseline LDL cholesterol levels modelled as 'untreated' (see text for methods)

**LDL cholesterol (mg/dl)**	**Primary prevention**			**Secondary prevention**		
	Percent statin use	Odds ratio (95% CI)	*P*-value	Percent statin use (%)	Odds ratio (95% CI)	*P*-value

<100	12.6% (11.4 to 13.9)	referent		39.0% (36.9 to 41.2)	referent	
≥100 and <130	14.8% (14.0 to 15.7)	1.20 (1.06 to 1.37)	0.005	38.2% (36.6 to 39.7)	0.97 (0.87 to 1.08)	0.54
≥130 and <160	16.2% (15.4 to 17.1)	1.34 (1.18 to 1.52)	<0.0001	34.4% (32.9 to 35.8)	0.82 (0.74 to 0.92)	0.0004
≥160	28.5% (27.5 to 29.5)	2.76 (2.45 to 3.11)	<0.0001	43.3% (41.9 to 44.7)	1.20 (1.08 to 1.33)	0.0009

*Proportions are described as percent and 95% confidence intervals for binomial distributions. Odds ratios with 95% confidence intervals were estimated by logistic regression analysis without adjustments.

### Statin treatment: multivariate logistic regression analysis

The parameters with the strongest association in bivariate analyses, namely presence of atherosclerotic disease and presence of hypertension were included in the model from the beginning. The best fit was achieved using the following covariates: age, age at first diagnosis of diabetes, smoking status, albuminuria, baseline LDL cholesterol, and BMI. Furthermore, the interaction terms 'sex*atherosclerotic disease', 'baseline LDL*atherosclerotic disease' and 'smoking status*atherosclerotic disease' were all highly significant. All parameters were independently associated with statin prescription, except for sex when the interaction term was used. The respective odds ratios, confidence intervals and *P*-values are shown in Table 5. Atherosclerotic disease, hypertension, former smoking, high LDL cholesterol, and albuminuria increased the odds for statin prescription, while it was decreased in higher or lower age, higher or lower age at diagnosis of diabetes, and higher or lower BMI. We found some significant interactions: the significant interaction term between atherosclerotic disease and sex indicated that women in secondary but not in primary prevention had lesser odds of receiving a statin. With this interaction term in place, sex by itself was not significant. The significant interaction between atherosclerotic disease and baseline LDL cholesterol indicated that high cholesterol was a stronger predictor of statin prescription in secondary than in primary prevention. The significant interaction between atherosclerotic disease and smoking status indicated that the increased odds of receiving a statin in former smokers were most pronounced in secondary prevention.

**Table 5:** Results of multivariate logistic regression analysis

**Covariate**	**Odds ratio**	**95% Confidence interval**	***P*-value**
Atherosclerotic disease	7.27	5.75 to 9.20	<0.0001
Hypertension	1.87	1.70 to 2.07	<0.0001
Smoking			0.08
Former smoker^a^	1.16	1.03 to 1.31	0.014
Current smoker^a^	1.03	0.93 to 1.31	0.58
Albuminuria (albumin ≥20 mg/dl)	1.06	1.003 to 1.16	0.037
Baseline LDL cholesterol^b^	1.11	1.06 to 1.16	<0.0001
Age at first diagnosis of diabetes^c^	0.93	0.90 to 0.96	<0.0001
Age^d^	0.86	0.82 to 0.90	<0.0001
Body mass index^e^	0.93	0.89 to 0.97	<0.0001

^a^In comparison to never smokers

^b^Odds ratio for an increase of 10 mg/dl. For details for modelling baseline LDL levels see text

^c^Odds ratio for an increase or a decrease of 10 years from the nadir, 56 years

^d^Odds ratio for an increase or a decrease of 10 years from the nadir, 66 years

^e^Odds ratio for an increase or a decrease of 5 kg/m^2 ^years from the nadir, 31.6 kg/m^2^

Using this model (total N = 37,110 patients), correct prediction of receiving a statin was 51% (sensitivity) and correct prediction of not receiving a statin was 77% (specificity). The overall proportion of correct prediction of our model was 70%.

## Discussion

Our results demonstrate that the presence of hypertension, high baseline LDL cholesterol, and microalbuminuria as well as a former smoking status, are positive predictors for receiving statin prescriptions. The most important predicting factor is the patient's cardiovascular risk. On the other hand, older or younger age, older or younger age at first diagnosis of diabetes, and higher or lower BMI are decreasing the odds of receiving a statin. Since patients with diabetes have a substantially increased risk of atherosclerotic vascular disease, identification of treatment for the prevention of vascular events is a public-health priority. The NCEP already in 2004 recommended LDL-C goals of < 100 mg/dl in high-risk patients, including patients with diabetes and an LDL-C of < 70 mg/dl as an option for very high risk patients [16]. However, statin prescription to every single diabetes patient is a controversially discussed issue. According to the recently published joint guidelines of the European Society of Cardiology (ESC) and the European Association for the Study of Diabetes (EASD) the goals of therapy proposed for diabetic patients in primary prevention are similar to those for non-diabetic patients with symptomatic CVD (total cholesterol < 174 mg/dl, LDL-C < 97 mg/dl) [3]. The decision whether statin therapy should be started in patients whose LDL-C is already < 100 mg/dl is left to individual judgement. In the Collaborative Atorvastatin Diabetes Study (CARDS), statins for primary prevention were investigated in patients with type 2 diabetes without high LDL-C and a significant reduction in major cardiovascular events, including stroke, was found [12]. Further studies confirmed that statins provide a wide range of cardiovascular risk benefit in patients with diabetes, independently of their baseline LDL cholesterol [24]. A meta-analysis in non-diabetic and diabetic patients suggested that diabetic patients, after adjustment for baseline characteristics, benefit even more than non-diabetes patients from lipid-lowering therapy in both primary and secondary prevention [14]. Overall, the evidence suggests that most diabetic patients should receive a statin.

The present study found that only 25% of patients with diabetes receive statin prescriptions. Prescription frequencies are higher in secondary prevention. Interestingly, in primary prevention, the odds of receiving a statin increased in parallel with the 5-year estimated risk for cardiovascular disease, indicating that prescription decisions are, at least in part, based on risk assessment. Previous studies in non-diabetic populations have shown that patients receiving statins are more likely to have severe cardiovascular comorbidities and to be elderly [25], but a remaining substantive underuse in high-risk patients has also been described [26]. Another interesting finding in our study was that both, the presence of hypertension and albuminuria were associated with increased odds of statin prescription, suggesting that both disease entities are being correctly perceived as predictors of CVD [27]. Surprisingly former smokers had higher odds of receiving a statin than never smokers or current smokers in secondary prevention. It could be postulated that former smokers (i) are considered to be still at a higher risk than never smokers and (ii) are 'rewarded', consciously or subconsciously, by their physicians for quitting smoking.

We identified three major parameters that were significantly associated with decreased statin prescription: young or old age, younger or older age at first diagnosis of diabetes, and subjects with higher or lower BMI values. Our findings that the elderly receive less treatment are in agreement with those of Teeling at al. [28], who showed a decreased probability of receiving a statin in patients age 65 and older, and to those of Ko et al. [29], who showed that in the elderly prescription of statins diminished progressively as CVD risk increased ("treatment-risk paradox"). In another study it was shown that prescription among patients > 74 years was 40% lower than in younger patients [30]. Interestingly, an inverted U-shaped curve association between age and statin prescription was described before [31]. Not only have several studies shown that elderly patients benefit from statin treatment if they have risk factors for vascular disease or established vascular disease [14,32], there is increasing evidence that treatment initiation early in life before the atherosclerotic process has become advanced may be beneficial [33]. The reasons behind fewer prescriptions at higher age may be concerns about treatment complications or shifting priorities for drug treatment from outcome-influencing drugs towards symptomatic treatments in multimorbid patients. Since age is the strongest component of the overall risk score and actual prescription decreases with higher age, the contribution of other risk factors to the odds of receiving statin prescriptions is even greater at higher age.

Our results of increasing statin use with increasing BMI are in accordance to findings of Agalliu et al. [34] and Neutel et al. [26]. However, these studies did not address individuals with BMI > 35 kg/m^2^. Our finding of decreased odds of statin prescription above a higher BMI and in normal weight individuals might reflect the physicians' belief that the former will not benefit from such treatment and the latter do not really need it.

We have previously shown that female sex is associated with statin undertreatment in patients with diabetes, especially in secondary prevention [17]. In the present study we confirm these results which are also in agreement to those of other investigators [31,35,36].

For estimating cardiovascular risk in primary prevention we used the newly developed risk equations from Sweden that were specifically designed and prospectively validated in patients with type 2 diabetes [20]. Other risk calculators have been criticized for not providing reliable estimates for such patients [37]. A further advantage of this risk calculator was that in the DUTY registry in more than 89% of the subjects all necessary parameters were available. Our finding showing an increase in the odds of receiving statin prescriptions in parallel to the increase in the overall 5-year estimated risk is reassuring, since it implies physicians' awareness of the concept of cardiovascular risk or individual risk estimation. The reasons for higher prescription rates in women in comparison to men in the mid-risk range (Figure 3) remain unclear.

The possible reasons behind the overall low prescription rates have to be addressed. Drug costs may play an important role in the wide underuse of statins. A study analyzing treatment costs at the time of data sampling of the DUTY registry found that costs for statins were higher than those for all other cardiovascular drugs that were prescribed to patients with type 2 diabetes together and the prescription rates for statins had been increasing 8-fold in the 10-year period before [23]. However, there is strong evidence to support that the statin-induced reduction of cardiovascular events in diabetic patients, both in primary and secondary prevention, is cost-effective [38]. More importantly, reluctant national recommendations, e. g. of disease management programmes, may play a major role [11].

### Limitations

Our study has several limitations. First, the list of the possible covariates examined is not complete. Statin prescription might depend on other factors not documented in the current registry. For example, the status of the patient's health insurance (private or statutory health insurance) as well as the socio-economic level of the subjects [39] may play a role. Second, physicians reported to the best of their knowledge of statin *prescription*; hence the data do not account for medication *dispensing *and *adherence *rates. Third, we don't have information about which statin at which dose was used. It is known that many statins are prescribed at doses lower than those shown to be effective in randomized trials [40]. And last, the degree of statin-induced lipid-lowering was calculated, not measured. The calculation of a mean lipid-lowering effect of 15% is only an approximate indicator but simulations with different effect sizes revealed similar results (data not shown).

To our knowledge this is the first study addressing the various predictors of statin prescription in patients with type 2 diabetes. The strength of our findings lies in the heterogeneity and the size of the population investigated and the use of multivariate analyses. By indentifying independent parameters that influence medical decision making in issues of vital importance, such as prescription of statins to patients with diabetes, these results represent a substantial opportunity for improvement in diabetes quality of care. The study contributes to understanding which parameters determine statin prescription but further studies are needed to investigate the reasons for *withholding *this drug therapy.

## Abbreviations

ADA: American Diabetes Association; BMI: body mass index; CVD: cardiovascular disease; CHD: coronary heart disease; LDL: low density lipoprotein; HDL: high density lipoprotein; DUTY: Diabetes mellitus needs unrestricted evaluation of patient data to yield treatment progress; HbA1c: hemoglobin-A1c; OR: odds ratio; CI: confidence interval; NCEP: National Cholesterol Education Program.

## Competing interests

HKB declares that there is no duality of interest associated with this manuscript. IGB has received honoraria for speaking engagements from Pfizer, Eli Lilly, MSD Sharp & Dohme and Essex Pharma. MB has received speakers' honoraria from AstraZeneca, Boehringer Ingelheim, Bristol Myers Squibb, MSD Sharp & Dohme, Essex Pharma, Pfizer, Servier and Sanofi-Aventis. He has been on an advisory panel for AstraZeneca, Boehringer Ingelheim, MSD Sharp & Dohme, Essex Pharma, Pfizer, Servier und Sanofi-Aventis. WK has received grant/research support from MSD Sharp & Dohme and speakers' honoraria from AstraZeneca, MSD Sharp & Dohme, Essex Pharma, Pfizer and Sanofi-Aventis. He has been on an advisory panel for MSD Sharp & Dohme and Essex Pharma. KPB is an employee of MSD Sharp & Dohme, Germany.

## Authors' contributions

HKB conceived of the study and its design, carried out the statistical analysis and interpretation of the data and has drafted the manuscript. IGB carried out the analysis and interpretation of the data and has been involved in drafting the manuscript. MB participated in the design of the registry and has been involved in critically revising the manuscript for important intellectual content. WK participated in the design of the registry and has been involved in critically revising the manuscript for important intellectual content. KPB conceived of the registry and participated in its coordination, acquisition of the data and acquisition of funding. All authors read and approved the final manuscript.

## Supplementary Material

Additional file 1 **Proportions and odds of statin prescription in bivariate analysis of various parameters**. Subjects were stratified according to primary of secondary prevention and groups were analyzed separately. Click here for file
