# Supplementary material for: Patterns and predictors of statin prescription in patients with type 2 diabetes
Source: Cardiovasc Diabetol. 2009 May 13;8:25. doi: 10.1186/1475-2840-8-25 (PMC2689197; doi:10.1186/1475-2840-8-25)
Supplement: Additional file 1 — Proportions and odds of statin prescription in bivariate analysis of various parameters. Subjects were stratified according to primary of secondary prevention and groups were analyzed separately. [file 1475-2840-8-25-S1.doc]

**Additional file 1:** Proportions and odds of statin prescription in bivariate analysis of various parameters*. Subjects were stratified according to primary of secondary prevention and groups were analyzed separately.

| **Characteristic** | **Primary prevention** |  |  | **Secondary prevention** |  |  |
| --- | --- | --- | --- | --- | --- | --- |
|  | Percent statin use | Odds ratio (95% CI) | P-value | Percent statin use | Odds ratio (95% CI) | P-value |
| **Number, %** | *n*=27,322  18.5%  (18.0 to 19.0) |  |  | *n*=17,571  38.1%  (37.4 to 38.9) | 2.72  (2.61 to 2.84)** | <0.0001 |
|  |  |  |  |  |  |  |
| **Sex (%)** |  |  |  |  |  |  |
| Male | 18.5%  (17.8 to 19.2) | referent |  | 41.3%  (40.3 to 42.3) | referent |  |
| Female | 18.5%  (17.8 to 19.1) | 0.996  (0.936 to 1.059) | 0.89 | 34.4%  (33.4 to 35.5) | 0.75  (0.70 to 0.79) | <0.0001 |
|  |  |  |  |  |  |  |
| **Age (years)** |  |  |  |  |  |  |
| <40 | 11.7%  (9.1 to 14.7) | referent |  | 37.8%  (22.5 to 55.2) | referent |  |
| ≥40 and <50 | 14.7%  (13.4 to 16.1) | 1.30  (0.98 to 1.72) | 0.0662 | 43.3%  (38.5 to 48.3) | 1.26  (0.63 to 2.51) | 0.52 |
| ≥50 and <60 | 18.9%  (18.0 to 19.9) | 1.77  (1.35 to 2.31) | <0.0001 | 43.3%  (41.1 to 45.4) | 1.25  (0.64 to 2.45) | 0.51 |
| ≥60 and <70 | 21.2%  (20.4 to 22.0) | 2.03  (1.56 to 2.64) | <0.0001 | 43.3%  (42.0 to 44.5) | 1.25  (0.64 to 2.44) | 0.51 |
| ≥70 and <80 | 17.9%  (16.9 to 18.9) | 1.64  (1.26 to 2.15) | 0.0003 | 36.8%  (35.6 to 38.1) | 0.96  (0.49 to 1.86) | 0.90 |
| ≥80 | 11.7%  (10.1 to 13.5) | 1.002  (0.74 to 1.36) | 0.99 | 21.9%  (20.1 to 23.7) | 0.46  (0.24 to 0.90) | 0.0234 |
|  |  |  |  |  |  |  |
| **Age at diagnosis of diabetes (years)** |  |  |  |  |  |  |
| <45 | 16.6%  (15.3 to 17.9) | referent |  | 44.8%  (41.8 to 47.8) | referent |  |
| ≥45 and <55 | 19.0%  (18.1 to 19.9) | 1.18  (1.06 to 1.32) | 0.003 | 42.1%  (40.5 to 43.9) | 0.90  (0.78 to 1.02) | 0.13 |
| ≥55 and <65 | 20.5%  (19.8 to 21.5) | 1.31  (1.18 to 1.45) | <0.0001 | 41.0%  (39.7 to 42.2) | 0.86  (0.75 to 0.98) | 0.0189 |
| ≥65 | 16.4%  (15.5 to 17.4) | 0.99  (0.88 to 1.11) | 0.86 | 31.8%  (30.6 to 33.0) | 0.58  (0.50 to 0.66) | <0.0001 |
|  |  |  |  |  |  |  |
| **Diabetes duration (years)** |  |  |  |  |  |  |
| <1 | 11.4%  (10.2 to 12.8) | referent |  | 37.9%  (34.3 to 41.6) | referent |  |
| ≥1 and <5 | 19.3%  (18.6 to 19.9) | 1.85  (1.62 to 2.10) | <0.0001 | 39.2%  (38.0 to 40.5) | 1.06  (0.90 to 1.24) | 0.50 |
| ≥5 and <10 | 18.9%  (17.9 to 19.9) | 1.80  (1.57 to 2.07) | <0.0001 | 38.0%  (36.6 to 39.4) | 1.002  (0.85 to 1.18) | 0.98 |
| ≥10 | 20.2%  (19.0 to 21.5) | 1.96  (1.70 to 2.27) | <0.0001 | 37.1%  (35.9 to 38.6) | 0.97  (0.83 to 1.14) | 0.72 |
|  |  |  |  |  |  |  |
| **Body mass index (kg/m^2^)** |  |  |  |  |  |  |
| <25 | 16.5%  (15.5 to 17.6) | referent |  | 33.0%  (31.5 to 34.7) | referent |  |
| ≥25 and <30 | 19.3%  (18.6 to 20.0) | 1.21  (1.11 to 1.32) | <0.0001 | 39.1%  (38.1 to 40.2) | 1.30  (1.20 to 1.42) | <0.0001 |
| ≥30 and <35 | 19.1%  (18.1 to 20.0) | 1.19  (1.08 to 1.32) | 0.0005 | 40.7%  (39.2 to 42.2) | 1.39  (1.27 to 1.53) | <0.0001 |
| ≥35 | 16.9%  (15.6 to 18.3) | 1.03  (0.91 to 1.16) | 0.65 | 37.6%  (35.0 to 40.2) | 1.22  (1.07 to 1.39) | 0.0028 |
|  |  |  |  |  |  |  |

**Additional file 1: cont’d**

| **Smoking status (%)** |  |  |  |  |  |  |
| --- | --- | --- | --- | --- | --- | --- |
| Never smoker | 18.1%  (17.5 to 18.7) | referent |  | 35.2%  (34.2 to 36.2) | referent |  |
| Former smoker | 20.1%  (19.0 to 21.3) | 1.14  (1.05 to 1.24) | 0.0014 | 44.8%  (43.4 to 46.2) | 1.49  (1.39 to 1.60) | <0.0001 |
| Current smoker | 18.5%  (17.4 to 19.6) | 1.03  (0.95 to 1.12) | 0.49 | 36.0%  (34.2 to 37.9) | 1.04  (0.95 to 1.14) | 0.42 |
|  |  |  |  |  |  |  |
| **Hypertension (%)** |  |  |  |  |  |  |
| No hypertension | 11.7%  (10.7 to 12.6) | referent |  | 22.9%  (20.2 to 25.8) |  |  |
| Hypertension | 19.9%  (19.3 to 20.4) | 1.88  (1.71 to 2.07) | <0.0001 | 39.0%  (38.2 to 39.7) | 2.15  (1.83 to 2.52) | <0.0001 |
|  |  |  |  |  |  |  |
| **HbA1c (%)** |  |  |  |  |  |  |
| <6.5 | 17.8%  (17.0 to 18.7) | referent |  | 36.9%  (35.5 to 38.4) | referent |  |
| ≥6.5 and <7.5 | 19.2%  (18.4 to 20.0) | 1.10  (1.01 to 1.18) | 0.021 | 39.8%  (38.5 41.0) | 1.13  (1.04 to 1.22) | 0.004 |
| ≥7.5 and <8.5 | 18.8%  (17.8 to 19.8) | 1.07  (0.98 to 1.17) | 0.16 | 36.7%  (35.3 to 38.2) | 0.99  (0.91 to 1.08) | 0.86 |
| ≥8.5 | 17.6%  (16.4 to 18.8) | 0.98  (0.89 to 1.09) | 0.74 | 38.5%  (36.7 to 40.3) | 1.07  (0.97 to 1.18) | 0.18 |
|  |  |  |  |  |  |  |
| **Kidney function** |  |  |  |  |  |  |
| No albuminuria | 17.6%  (17.0 to 18.2) | referent |  | 37.5%  (36.4 to 38.6) | referent |  |
| Microalbuminuria  (≥20 mg/l) | 19.6%  (18.7 to 20.4) | 1.14  (1.06 to 1.22) | <0.0001 | 39.1%  (38.0 to 40.3) | 1.07  (1.003 to 1.15) | 0.04 |
|  |  |  |  |  |  |  |
| **Lipoprotein concentrations (mg/dl)** |  |  |  |  |  |  |
| Total cholesterol |  |  |  |  |  |  |
| <200 | 19.9%  (19.0 to 20.9) | referent |  | 47.2%  (45.9 to 48.5) | referent |  |
| ≥200 and <240 | 16.2%  (15.5 to 16.9) | 0.78  (0.72 to 0.84) | <0.0001 | 34.8%  (33.6 to 36.1) | 0.60  (0.55 to 0.64) | <0.0001 |
| ≥240 | 19.7%  (18.9 to 20.5) | 0.98  (0.91 to 1.06) | 0.65 | 32.6%  (31.4 to 33.9) | 0.54  (0.50 to 0.58) | <0.0001 |
|  |  |  |  |  |  |  |
| LDL cholesterol |  |  |  |  |  |  |
| <100 | 23.8%  (22.4 to 25.3) | referent |  | 54.9%  (53.0 to 56.7) | referent |  |
| ≥100 and <130 | 19.7%  (18.8 to 20.7) | 0.78  (0.71 to 0.87) | <0.0001 | 42.0%  (40.6 to 43.6) | 0.60  (0.54 to 0.66) | <0.0001 |
| ≥130 and <160 | 16.9%  (16.1 to 17.8) | 0.65  (0.59 to 0.72) | <0.0001 | 32.0%  (30.6 to 33.5) | 0.39  (0.35 to 0.43) | <0.0001 |
| ≥160 | 19.0%  (18.0 to 20.0) | 0.75  (0.68 to 0.83) | <0.0001 | 30.9%  (29.5 to 32.5) | 0.37  (0.33 to 0.41) | <0.0001 |
|  |  |  |  |  |  |  |
| HDL cholesterol |  |  |  |  |  |  |
| <40 (M) or  <50 (F) | 19.9%  (19.1 to 20.7) | referent |  | 38.2%  (37.0 to 39.3) | referent |  |
| ≥40 (M) or  ≥50 (F) | 19.1  (18.5 to 19.7) | 1.05  (0.988 to 1.12) | 0.11 | 39.9%  (38.9 to 40.9) | 0.93  (0.87 to 0.99) | 0.0226 |
|  |  |  |  |  |  |  |
| Triglycerides |  |  |  |  |  |  |
| <150 | 9.8%  (9.2 to 10.5) | referent |  | 37.3%  (36.0 to 38.7) | referent |  |
| ≥150 and <400 | 20.0%  (19.3 to 20.7) | 1.29  (1.20 to 1.38) | <0.0001 | 38.9%  (37.9 to 40.0) | 1.07  (0.999 to 1.15) | 0.054 |
| ≥400 | 22.9%  (20.6 to 25.3) | 1.53  (1.32 to 1.77) | <0.0001 | 45.4%  (42.0 to 48.8) | 1.40  (1.21 to 1.62) | <0.0001 |

**Additional file 1: cont’d**

| **Estimated 5-year cardiovascular risk (%)** |  |  |  |  |  |  |
| --- | --- | --- | --- | --- | --- | --- |
| <5 | 6.6%  (5.7 to 7.5) | referent |  |  |  |  |
| ≥5 and <10 | 13.9%  (13.0 to 14.8) | 2.29  (1.95 to 2.69) | <0.0001 |  |  |  |
| ≥10 and <15 | 19.7%  (18.6 to 20.8) | 3.48  (2.96 to 4.08) | <0.0001 |  |  |  |
| ≥15 and <20 | 23.4%  (20.1 to 24.8) | 4.34  (3.68 to 5.1) | <0.0001 |  |  |  |
| ≥20 and <25 | 23.9%  (22.4 to 25.6) | 4.45  (3.75 to 5.28) | <0.0001 |  |  |  |
| ≥25 and <30 | 26.9%  (24.5 to 28.9) | 5.16  (4.3 to 6.19) | <0.0001 |  |  |  |
| ≥30 | 26.9%  (25.1 to 28.8) | 5.23  (4.4 to 6.22) | <0.0001 |  |  |  |
|  |  |  |  |  |  |  |
| **Atherosclerotic complications** |  |  |  |  |  |  |
| Coronary heart disease |  |  |  | 40.1%  (39.3 to 40.9) | 2.69  (2.57 to 2.80)** | <0.0001 |
| Cerebrovascular disease |  |  |  | 37.3%  (35.8 to 38.9) | 1.83  (1.71 to 1.97)** | <0.0001 |
| Peripheral arterial occlusive disease |  |  |  | 38.0%  (36.6 to 39.4) | 1.94  (1.82 to 2.07)** | <0.0001 |

*Proportions are described as percent and 95% confidence intervals for binomial distributions. Odds ratios with 95% confidence intervals were estimated by logistic regression analysis without adjustments.

**in comparison to primary prevention.
